# Aberrant methylation and downregulation of ZNF667-AS1 and ZNF667 promote the malignant progression of laryngeal squamous cell carcinoma

**DOI:** 10.1186/s12929-019-0506-0

**Published:** 2019-01-26

**Authors:** Wenxia Meng, Weina Cui, Lei Zhao, Weiwei Chi, Huan Cao, Baoshan Wang

**Affiliations:** 1 Department of Otorhinolaryngology, The Second Hospital of Hebei Medical University, 215 Heping West Road, Shijiazhuang, 050000 Hebei China; 2 Department of Otorhinolaryngology, The Affiliated Hospital of Hebei University, Baoding, 071000 Hebei China; 3 Department of Otorhinolaryngology, The First Hospital of Hebei Medical University, Shijiazhuang, 050031 Hebei China

**Keywords:** Laryngeal squamous cell carcinoma, Methylation, ZNF667-AS1, ZNF667

## Abstract

**Background:**

Dysregulated long noncoding RNAs (lncRNAs) are involved in the development of tumor. Aberrant methylation is one of the most frequent epigenetic alterations that regulate the expression of genes. The aim of this study was to determine the expression and methylation status of ZNF667-AS1 and ZNF667, elucidate their biological function in the development of LSCC, and identify a cis-regulation of ZNF667-AS1 to ZNF667.

**Methods:**

The expression and methylation status of ZNF667-AS1 and ZNF667 in laryngeal cancer cell lines and LSCC samples were tested respectively. The function of two laryngeal cancer cell lines with overexpression of ZNF667-AS1 or ZNF667 was detected. The regulation between ZNF667-AS1 and ZNF667 was determined.

**Results:**

Significant downregulation of ZNF667-AS1 was detected in laryngeal cancer cell lines and LSCC tumor tissues. The reduced expression of ZNF667-AS1 was associated with moderate/poor pathological differentiation of LSCC tumor tissues. Aberrant hypermethylation of the CpG sites of ZNF667-AS1, closing to the transcriptional start site (TSS), was more critical for gene silencing, and associated with moderate/poor pathological differentiation. In vitro hypermethylation of promoter region closing to TSS of ZNF667-AS1 decreased the luciferase reporter activity. Overexpression of ZNF667-AS1 reduced the proliferation, migration, and invasion ability of AMC-HN-8 and TU177 cells. The sense strand, ZNF667, was positively correlated with ZNF667-AS1 in expression and function. Overexpression of ZNF667-AS1 led to increased expression of ZNF667 in mRNA and protein level. ZNF667-AS1 and ZNF667 may be associated with epithelial-mesenchymal transition (EMT) process.

**Conclusions:**

ZNF667-AS1 and ZNF667 are both down-regulated by hypermethylation, and they serve as tumor suppressor genes in LSCC. ZNF667-AS1 regulates the expression of ZNF667 in cis.

**Electronic supplementary material:**

The online version of this article (10.1186/s12929-019-0506-0) contains supplementary material, which is available to authorized users.

## Background

Laryngeal squamous cell carcinoma (LSCC) is one of the most common types of laryngeal cancer. According to Global Cancer Statistics 2018, there are 177,422 new cases of laryngeal cancer worldwide in 2018, which presents 1% of all new cancer cases [1]. The death of Laryngeal cancer is 94,771 cases worldwide, 1% of all sites cancer [1]. In 2012, there were 157,000 new cases and 83,000 death cases of LSCC worldwide [2]. The mortality of LSCC has significantly increased over the past decades worldwide. It is urgently necessary for early detection of LSCC and new methods of treatment.

**Fig. 1:**
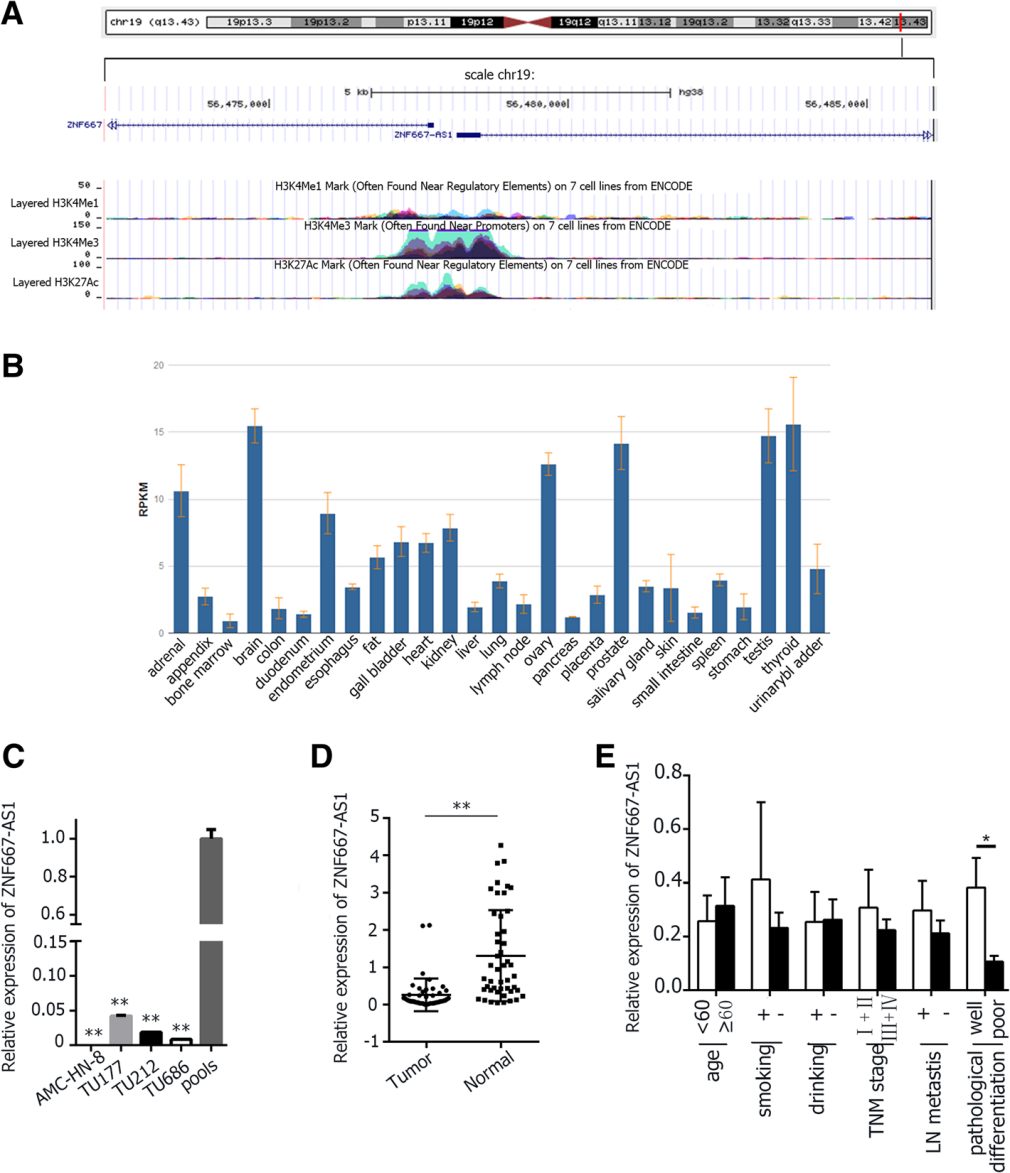
The expression of ZNF667-AS1. **a**. Location of ZNF667-AS1, cited from UCSC. **b**. The expression level of ZNF667-AS1 in various normal tissues, cited from NCBI. **c**. Relative expression of ZNF667-AS1 in four laryngeal cancer cell lines. **d**. Relative expression of ZNF667-AS1 in tumor tissues and corresponding LSCC normal tissues. **e**. Relative expression of ZNF667-AS1 in different subgroups. Data were mean ± S.D. of three independent determinations. Student’s t-test was used for *P* value assessment. *P < 0.05, **P < 0.01

Long noncoding RNAs (lncRNAs), more than 200 nucleotides in length, have diverse functions [3], and play important roles in the development of tumor, such as lncRNA RP11-169D4.1, HOTAIR, NEAT1, and H19 [4–7], and so on. LSCC patients with high expression level of HOTAIR were in advanced clinical stage or with poor pathological differentiation [5]. Higher NEAT1 expression in LSCC was associated with advanced clinical stage and metastasis [6]. The high expression level of H19 was correlated with low survival rate of LSCC patients [7]. Detecting the lncRNAs in LSCC by microarray, we found the expression level of ZNF667-AS1 (also known as MORT [8]) was greatly reduced in tumor tissues compared to corresponding normal tissues. We further verified it by quantitative real-time polymerase chain reaction (qRT-PCR). ZNF667-AS1, a 1837 bp polyadenylated lncRNA, is located on 19q13.43 (GRCh 38/hg38 database, chr19: 56477874 -56495436, NCBI: NR_036521.1) (Fig. 1a), and has a large CpG island ranging from -1027 to + 540 bp determined by MethPrimer program (Fig. 3a). Increasing evidence has emerged to suggest that lncRNA ZNF667-AS1 is down-regulated in various tumors [8–11]. However, the expression of ZNF667-AS1 in LSCC remains unknown.

lncRNAs can act near their site of synthesis to regulate the expression of other genes in cis (i.e., on the same chromosome) or act in trans (i.e., across multiple chromosomes). ZNF667-AS1 and ZNF667 are head-to-head antisense-sense strands. ZNF667 is also located on 19q13.43 (GRCh 38/hg38 database, chr19: 56439324- 56,477,401, NCBI: NM_022103) (Fig. 1a). Both of the genes are at a distance of 473 bp from each other. Determined by MethPrimer program, ZNF667-AS1 and ZNF667 have the same CpG islands in their promoters. ZNF667 (also known as Mipu1), a C2H2-type zinc finger transcription factor, lies in cell nucleus. ZNF667 was suggested to play an important role in oxidative stress [12], and was an anti-apoptotic factor in some conditions [13, 14]. Recently, it was reported that ZNF667 served as a putative oncogene in human hepatocellular carcinoma [15]. The expression of ZNF667 in LSCC is still unknown.

DNA methylation is one of the epigenetic alterations identified in cancer. Hypermethylation in CpG islands of promoters can lead to silence of tumor suppressor genes. There are some reports about aberrant DNA methylation in LSCC [5, 16, 17]. Hypermethylation of PTEN led to high expression level of oncogenic HOTAIR in LSCC [5]. In male LSCC patients, elevated CMTM3 methylation was a risk factor [16]. Tumor suppressor gene MYCT1 was hypermethylated and down-regulated in LSCC [17]. Given the large CpG islands of ZNF667-AS1 and ZNF667, we hypothesized that aberrant hypermethylation may be one of the mechanisms in ZNF667-AS1 and ZNF667 inactivation in LSCC. In the present study, we detected the expression and methylation status of ZNF667-AS1 and ZNF667 in laryngeal cancer cell lines and LSCC tissues, elucidated the role of ZNF667-AS1 and ZNF667 in the pathogenesis of LSCC, and further identified the correlation between ZNF667-AS1 and ZNF667.

## Materials and methods

### Patients and specimens

Forty-seven LSCC patients with tumor tissues and corresponding adjacent normal tissues were enrolled from the Second Hospital of Hebei Medical University between the years of 2016 and 2018. All procedures performed in this study were in accordance with the ethical standards of the institutional research committee and with the 2008 Helsinki declaration. The study was approved by the Ethics Committee of Hebei Medical University and the Second Hospital of Hebei Medical University. Written informed consent was obtained from all study subjects. The patients were all males with a median age of 61 years (ranged from 44 to 78 years). All of the tissues were frozen and stored at − 80 °C in the Biobank of Otorhinolaryngology Head and Neck Surgery of Hebei Medical University to extract genomic DNA and RNA. The clinical characteristics were obtained from hospital recordings and pathological diagnosis.

### Cell culture and treatment

Four laryngeal cancer cell lines (AMC-HN-8, TU177, TU212, and TU686) were cultured in appropriate medium with 10% FBS in CO_2_ incubator (Mod: 371, Thermo Fisher, USA) at 37 °C, 5% CO_2_. All of the four cell lines were treated with DNA methyltransferase inhibitor 5-aza-2′-deoxycytidine (5-Aza-dC) in the concentration of 7.5 μmol/L for 72 h. Control cells received no drug treatment. The cells were then harvested for DNA and RNA extraction.

### ZNF667-AS1 and ZNF667 expression by qRT-PCR

The total RNAs were obtained from the tissues and cell lines by Eastep®Super Total RNA Extraction Kit (Promega, USA). Using Transcriptor First Strand cDNA Synthesis Kit (Roche, Germany) reverse transcription was done to change the RNA to cDNA. All primers and reaction conditions were listed in Additional file 1: Table S1. The quantitative real-time RT-PCR was performed with GoTaq®qPCR Master Mix (Promega, USA). The relative expression levels were calculated with the method of 2^-∆∆Ct^ and the glyceraldehyde-3-phosphate dehydrogenase (GAPDH) was adopted as an internal control. All the samples were run in triplicate.

### Methylation analysis of ZNF667-AS1 and ZNF667 via bisulfite genomic sequencing (BGS) method

Among 47 pairs of samples with RNA, the DNA from 32 pairs of samples were obtained, and were treated with bisulfite using Epitect Fast Bisulfite Conversion Kits (Qiagen, Germany) according to the manufacturer’s instructions. The methylation status of every CpG site in the CpG islands of ZNF667-AS1 and ZNF667 was examined by BGS assay in four laryngeal cancer cell lines and two pairs of LSCC tissues. Primers of BGS, recognizing sodium bisulfite converted DNA, were designed based on three CpG island regions of ZNF667-AS1 (CpG island region 1: from − 844 to − 395 bp relative to the transcriptional start site, CpG island region 2: from − 200 to + 71 bp, CpG island region 3: from + 277 to + 547 bp) and three CpG island regions of ZNF667 (CpG island region 3: from − 1000 to − 749 bp, CpG island region 2: from − 482 to − 275 bp, CpG island region 1: from + 146 to + 524 bp). All primers and reaction conditions were listed in Additional file 1: Table S1. Twenty-five nanogram of bisulfite-modified DNA was subjected to PCR amplification and the PCR products were cloned into pGEM-T easy vectors (Promega, USA) and 8–10 clones of each specimen were sequenced by automated fluorescence-based DNA sequencing. Percentage methylation was determined as percentage of methylated cytosines from 8 to 10 sequenced colonies.

### Methylation analysis of ZNF667-AS1 and ZNF667 via methylation-specific polymerase chain reaction (MSP) method

The methylation status of ZNF667-AS1 and ZNF667 in four laryngeal cell lines and 32 pairs of LSCC tissues were determined by MSP method. Regions of MSP were located in three CpG island regions of ZNF667-AS1 (region 1 from − 925 to -744 bp, region 2 from − 158 to -4 bp, region 3 from + 326 to + 479 bp) and three CpG island regions of ZNF667 (region 1 from + 276 to + 453, region 2 from − 469 to -316 bp, region 3 from − 951 to − 791). MSP primers of Region 1, 2 and 3 of ZNF667 were reverse complement to that of ZNF667-AS1 respectively. All primers and reaction conditions were listed in Additional file 1: Table S1. Bisulfite-modified DNA was used as PCR template, and the products were analyzed on 2% agarose gel. If a visible band was observed in the methylation reaction, the DNA was determined to be methylated. If a visible band was observed in the unmethylation reaction, it was determined to be unmethylated. If a band was visible in both methylation and unmethylation reaction, it was determined to be partly methylated.

### Luciferase reporter assay

According to the CpG island regions, five promoter fragments of ZNF667-AS1 and ZNF667 were chosen as luciferase reporter genes for inserting to pGL3-basic plasmid. The three reporter genes of ZNF667-AS1 were located in three CpG island regions (pGL3-region 1 + 2: from − 636 to + 52 bp, pGL3-region 2: from − 173 to + 52 bp, pGL3-region 3: from + 53 to + 545 bp), the two reporter genes of ZNF667 were located in CpG island region 2 (pGL3-region 2: from − 543 to − 275 bp comprising the same CpG sites as that of ZNF667-AS1, pGL3-region 4: from − 270 to + 21 bp). All primers and reaction conditions were listed in Additional file 1: Table S1. DNA was used as PCR templates, and the product was inserted to pGEM-T easy vector. The vectors with correct fragments verified by sequencing were digested with HindIII and BglII (Thermo Scientific, USA). The inserts were devided into two parts, one part was treated with CpG Methyltransferase (M.SssI) (New England Biolabs, USA) overnight, the other remained untreated. The methylated and unmethylated DNA fragments were ligated respectively to the pGL3-basic luciferase vectors. One hundred and fifty nanogram (ng) plasmids were transiently cotransfected with fifty ng pRL-TK into 293 T cells, and then incubated at 37 °C for 48 h. Luciferase activity was measured with the Dual-Luciferase Reporter Assay System (Promega, USA). Promoter activities were expressed as the ratio of Firefly luciferase to Renilla luciferase activity.

### Gene transfection

For overexpression of ZNF667-AS1 or ZNF667, the sequence of ZNF667-AS1 or ZNF667 was synthesized and subcloned into pcDNA3.1 (Invitrogen, USA). AMC-HN-8 and TU177 cells were transfected respectively with 3μg pcDNA3.1-ZNF667-AS1 or pcDNA3.1-ZNF667 using 6ul Lipofectamine®2000 reagent (Invitrogen, USA) according to the manufacture’s instruction. AS control, the pcDNA3.1 empty vector was transfected at the same time.

### Cell proliferation assay

The proliferation ability of transfected laryngeal cancer cell lines AMC-HN-8 and TU177 were measured with MTS assay. After 24 h of the transfection with pcDNA3.1, pcDNA3.1-ZNF667-AS1, or pcDNA3.1-ZNF667, 2 × 10^3^ AMC-HN-8 or TU177 cells/well were suspended in 100 μl culture medium with 10% FBS and seeded into 96-well plate. Following the specifications of CellTiter96®AQ_ueous_One Solution Cell Proliferation Assay Kit (Promega, USA), 20 μl MTS/well was added into 96-well plate and incubated for 2 h. The absorbance values of each well were detected at a wavelength of 490 nm by Spark® multimode microplate reader (Mod: SPARK 10 M, TECAN, Switzerland).

### Plate colony formation assay

2000 laryngeal cancer cells AMC-HN-8 or TU177 transfected with pcDNA3.1, pcDNA3.1-ZNF667-AS1, or pcDNA3.1-ZNF667 were seeded into each well of a 6-well plate and incubated at 37 °C for 10 days. The colonies were fixed and stained in a dye solution containing 1% crystal violet, and the number of colonies formed with more than 50 cells was counted.

### In vitro cell migration and invasion assay

A 24-well plate containing 8-μm-pore size chamber inserts (Corning Costar, USA) was used to evaluate the migration and invasion of cancer cells transfected with pcDNA3.1- ZNF667-AS1, pcDNA3.1- ZNF667, or pcDNA3.1. For the invasion assay, the membrane was coated with Matrigel to form a matrix barrier. 1 × 10^5^ Cells in 100 μl FBS-free culture medium were loaded onto each filter insert (upper chamber) and 650 ul of culture medium with 10% FBS was added in each lower chamber, then incubated at 37 °C for 24 h or 48 h, respectively for migration assay and invasion assay. After harvested, the bottom of the insert was fixed and stained with crystal violet. The number of cells that had migrated or invaded was counted in five randomly selected microscopic fields.

### Subcellular fractionation

To isolate RNA from nucleus or cytoplasm of cell lines, the PARIS™ Kit Protein and RNA Isolation System (Invitrogen, USA) was used following the manufacturer’s instructions. GAPDH and U6 were respectively used as cytoplasmic and nuclear control.

### Western blot analysis

Protein was extracted from pcDNA3.1 or pcDNA3.1-ZNF667-AS1 transfected AMC-HN-8 or TU177 cells using RIPA lysis buffer added with PMSF (Solarbio, China) and protease inhibitor cocktail (Promega, USA), and followed by ultrasonication (Thermo FB120, USA). The protein concentration was determined using the BCA Protein Assay Kit (Generay, China). Protein lysates were mixed with SDS-PAGE loading buffer (Solarbio, China) and heated at 99 °C for 5 min. Twenty ug of protein was separated by SDS-PAGE and transferred onto PVDF membranes (Bio-Rad, USA). The transferred membranes were blocked with 5% skim milk (BD, USA) at room temperature for 2 h. Blots were incubated with Rabbit anti-human ZNF667 polyclonal antibody (Gene Tex, USA, 1:1000) or GAPDH (Proteintech, USA, 1:5000) overnight at 4 °C, and then incubated with horseradish peroxidase- conjugated secondary antibody at room temperature for 2 h. The band was visualized with enhanced chemiluminescence (ECL) detection reagents (vazyme, China) using ChemiDoc™ XRS+ (Bio-Rad, USA).

### Data mining

The GEPIA (http://gepia.cancer-pku.cn/), Gene Expression Profiling Interactive Analysis, is a newly developed interactive web server for analyzing the RNA sequencing expression data of 9736 tumors and 8587 normal samples from the TCGA and GTEx projects, using a standard processing pipeline [18]. It provides tumor and normal differential expression analysis, survival plots, and so on.

### Statistical analysis

Statistical analysis was performed by SPSS21.0 software (SPSS, Chicago, IL, USA). The qRT-PCR results were expressed as the mean ± S.D., and the difference between different groups was analyzed by Student’s t test. The status of gene methylation between different groups was analyzed using Chi-square test. The correlation between ZNF667-AS1 and ZNF667 was tested by Spearman correlation analysis. All statistical tests were two sided; and *P* < 0.05 was considered significant. Related diagrams were completed with GraphPad Prism7.0 (GraphPad Software Inc., La Jolla California USA).

## Results

### Silencing of ZNF667-AS1 in laryngeal cancer cell lines and LSCC tissues

As indicated by NCBI, ZNF667-AS1 was expressed at different level in different normal tissues (Fig. 1b). As shown in Fig. 1c and d, the expression level of ZNF667-AS1 was remarkably reduced or silenced in four laryngeal cancer cell lines and LSCC tissues. In 47 tumor tissues, the decreased expression of ZNF667-AS1 was associated with moderate/poor pathological differentiation (Fig. 1e).

### Silencing of ZNF667 in laryngeal cancer cell lines and LSCC tissues

**Fig. 2:**
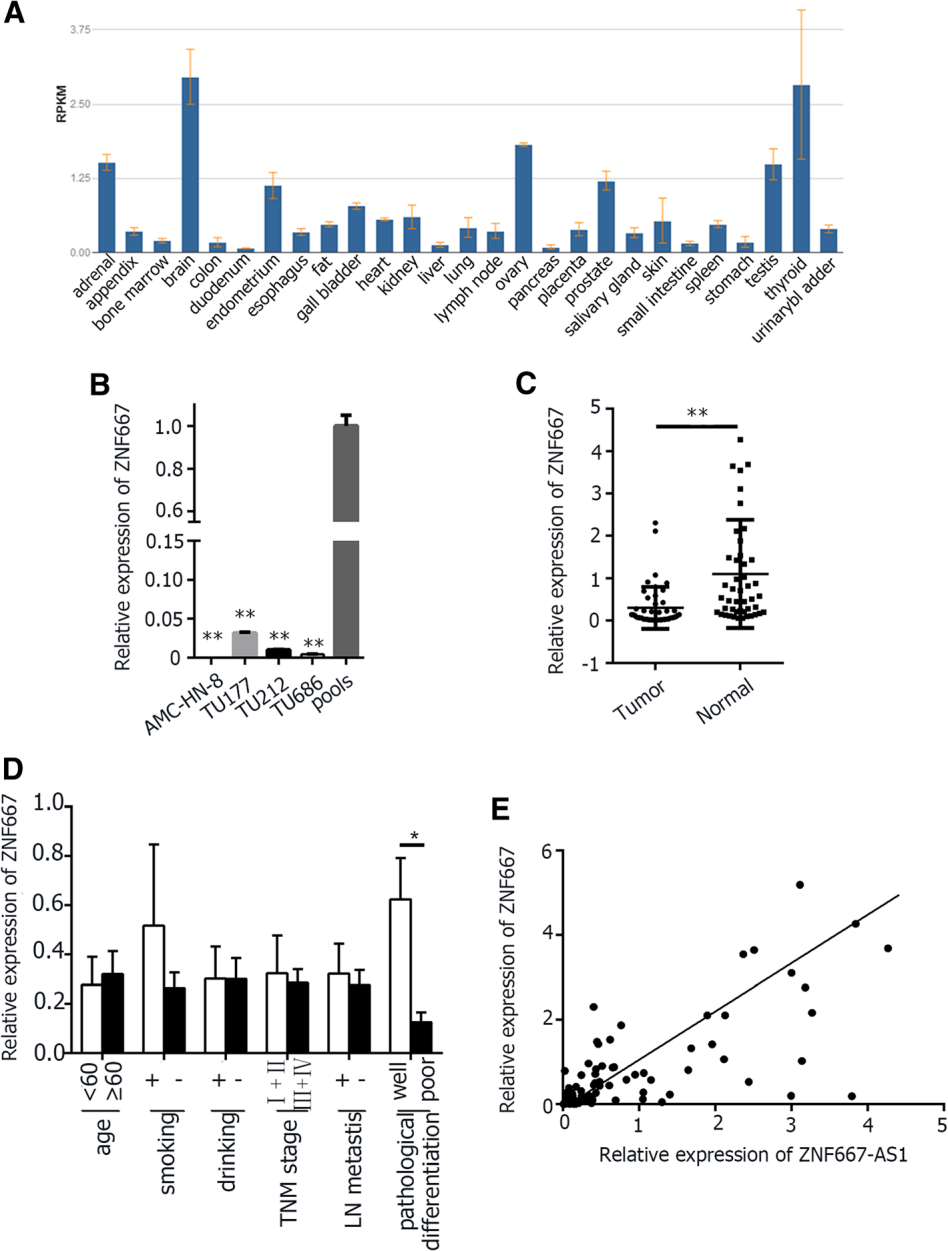
The expression of ZNF667. **a**. The expression level of ZNF667 in various normal tissues, cited from NCBI. **b**. Relative expression of ZNF667 in laryngeal cancer cell lines. **c**. Relative expression of ZNF667 in tumor tissues and corresponding LSCC normal tissues. **d**. Relative expression of ZNF667 in different subgroups. **e**. The correlation of ZNF667-AS1 and ZNF667, r = 0.7321, *p* < 0.01. Data were mean ± S.D. of three independent determinations. Student’s t-test was used for P value assessment. *P < 0.05, **P < 0.01

As indicated by NCBI, ZNF667 was also expressed at different level in different normal tissues (Fig. 2a). As shown in Fig. 2b and c, the expression level of ZNF667 was also remarkably reduced or silenced in four laryngeal cancer cell lines and tumor tissues. In 47 tumor tissues, the decreased expression level of ZNF667 was associated with moderate/poor pathological differentiation (Fig. 2d).

The expression level of ZNF667-AS1 and ZNF667 in 47 pairs of LSCC tissues were further analyzed by correlation analysis, and they had positive correlations, r = 0.7321, *p* < 0.01, as shown in Fig. 2e. Therefore, ZNF667-AS1 and ZNF667 might be under the same regulation and be functionally linked.

### Up-regulation of ZNF667-AS1 and ZNF667 by 5-Aza-dC treatment in laryngeal cancer cell lines

**Fig. 3:**
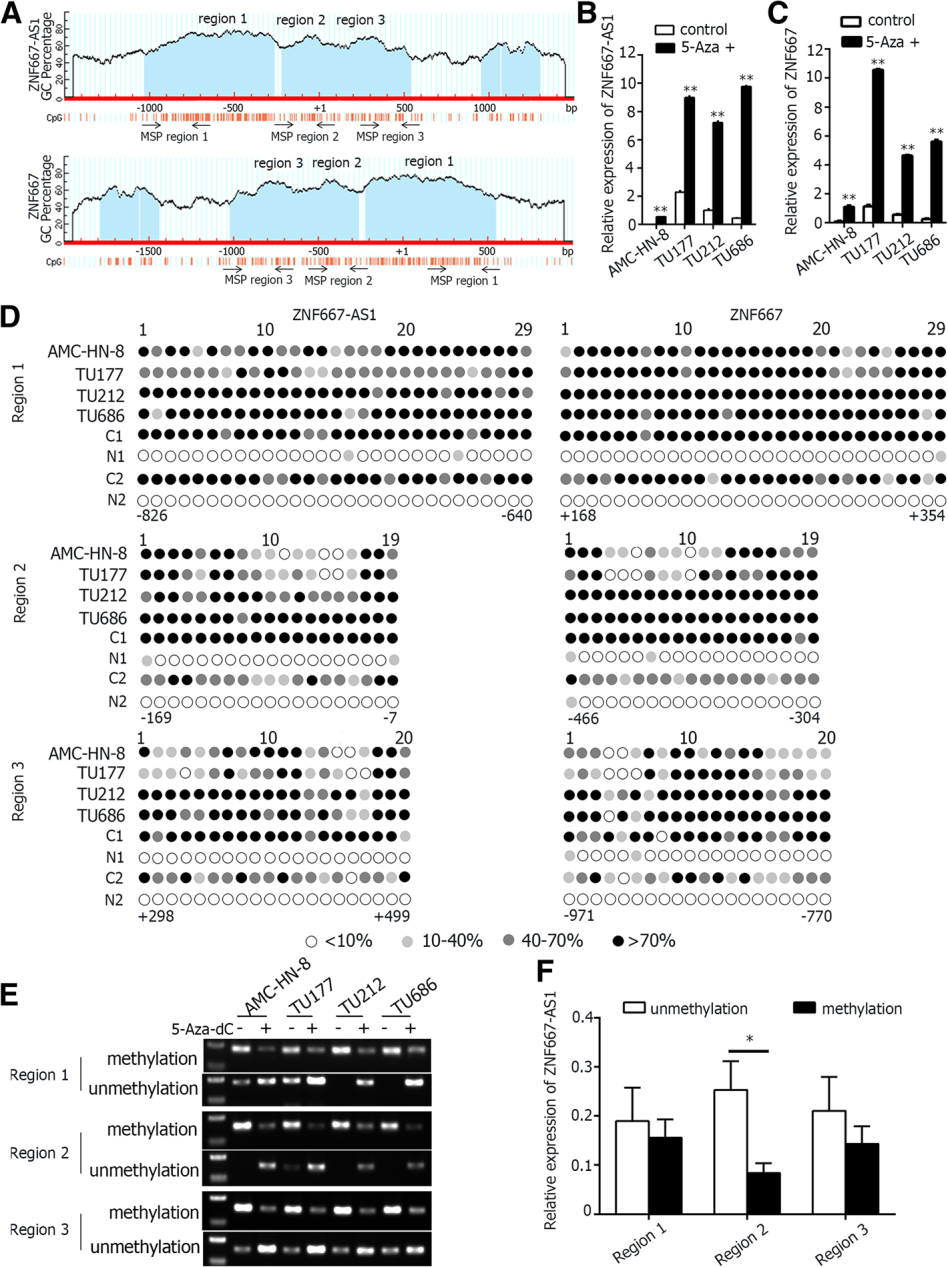
Methylation status of ZNF667-AS1 and ZNF667. **a**. Schematic structure of CpG islands of ZNF667-AS1 and ZNF667, cited from MethPrimer. **b**. The expression of ZNF667-AS1 increased after treatment with 5-Aza-dC in four laryngeal cancer cell lines. **c**. The expression of ZNF667 increased after treatment with 5-Aza-dC in four laryngeal cancer cell lines. **d**. High-resolution mapping of the methylation status of every CpG site in ZNF667-AS1 and ZNF667 CpG islands in four laryngeal cancer cell lines and two pairs of LSCC tissues determined by BGS. Each CpG site is shown at the top row as an individual number. The color of circles for each CpG site represented the percentage of methylation. **e**. The methylation status of three regions of ZNF667-AS1 detected by MSP analysis in four cancer cell lines with or without 5-Aza-dC treatment. **f**. Relative expression of ZNF667-AS1 in the tumor tissues with and without methylation of the three regions, which was expressed as mean ± S.D. * *P* < 0.05

As shown in Fig. 3a, ZNF667-AS1 and ZNF667 had the same large CpG islands regardless of the reverse direction. As shown in Fig. 3b and c, after treatment with DNA methyltransferase inhibitor 5-Aza-dC, the expression levels of ZNF667-AS1 and ZNF667 were all increased in laryngeal cancer cell lines, indicating the important role of DNA methylation in silencing the expression of ZNF667-AS1 and ZNF667. To further prove, frequent hypermethylation of the CpG islands of ZNF667-AS1 and ZNF667 were detected by bisulfite genomic sequencing (BGS) in four laryngeal cancer cell lines and two pairs of LSCC tissues. As shown in Fig. 3d, region 1, 2, and 3 of CpG island of cancer cell lines had identical hypermethylation pattern in the promoters of ZNF667-AS1 and ZNF667. Importantly, two pairs of LSCC tissues, chose according to the RNA expression level, had similar BGS results. In tumor tissues, the sites of CpG island were hypermethylated, and in normal tissues, the sites of CpG island were almost unmethylated.

### Expression level of ZNF667-AS1 was associated with methylation status in CpG region closing to the transcriptional start site

As shown in Fig. 3e, AMC-HN-8, TU177, TU122 and TU686 were all methylated in three regions of CpG island, indicated by MSP. In region 2, CpG sites were mainly fully methylated except for TU177, while in region 3, CpG sites were mainly partly methylated. As shown in Fig. 3f, in 32 tumor tissues with both RNA and DNA, samples with methylation in region 2 displayed significant lower expression level of ZNF667-AS1 (*P* < 0.05). In both region 1 and region 3, the methylation had no correlation with expression. As shown in Table 1, hypermethylation of region 2 was associated with moderate/poor pathological differentiation in 32 tumor tissues. Comparing the tumor tissues with normal tissues of those 32 pairs of LSCC tissues, the rate of hypermethylation was significantly higher in tumor tissues in all of the three regions of CpG island (Table 1).

**Table 1:** Methylation status of ZNF667-AS1 in LSCC tissues

Group	N	Methylation frequency					
		Region 1		Region 2		Region 3	
		*n* (%)	*P*	*n* (%)	*P*	*n* (%)	*P*
Age							
<60	13	8 (61.5)	1.0	8 (61.5)	0.473	10 (76.9)	0.2673
≥ 60	19	12 (63.2)		8 (42.1)		10 (52.6)	
Smoking							
Positive	27	18 (66.7)	0.338	14 (51.9)	1.0	18 (66.7)	0.338
Negative	5	2 (40.0)		2 (0.4)		2 (40.0)	
Drinking							
Positive	19	11 (57.9)	0.713	9 (47.4)	1.0	14 (73.7)	0.150
Negative	13	9 (69.2		7 (53.8)		6 (46.2)	
Pathological differentiation							
Well	15	9 (60.0)	1.0	4 (26.7)	0.032	7 (46.7)	0.144
Moderate/poor	17	11 (64.7)		12 (70.6)		13 (76.5)	
TNM stage							
I + II	14	11 (78.6)	0.147	8 (57.1)	0.722	10 (71.4)	0.471
III + IV	18	9 (50.0)		8 (44.4)		10 (55.6)	
LN metastasis							
Positive	12	7 (58.3)	0.734	7 (58.3)	0.716	7 (58.3)	0.724
Negative	20	13 (65.0)		9 (45.0)		13 (65.0)	
Tissues							
Tumor	32	20 (62.5)	< 0.001	16 (50.0)	< 0.001	20 (62.5)	< 0.001
Normal	32	3 (9.3)		1 (3.1)		4 (12.5)	

In 32 tumor tissues, hypermethylation of ZNF667-AS1 was associated with moderate/poor pathological differentiation. Comparing the tumor tissues with normal tissues of LSCC, the rate of hypermethylation was significantly higher in tumor tissues in all of the three regions of CpG island

### Expression level of ZNF667 was associated with methylation status in CpG region closing to the transcriptional start site

In 32 pairs of LSCC tissues, we detected the methylation pattern of ZNF667 by MSP. The representative MSP in patients was shown in Additional file 2: Figure S1A. Similarly, the methylation of region 2 in ZNF667 promoter was correlated with low expression (*P* < 0.05), and the others had no correlation (Additional file 2: Figure S1B). Region 2 was closing to the transcriptional start site than the others.

### In vitro hypermethylation of the promoter of ZNF667-AS1 or ZNF667 led to a significant decrease in luciferase activity respectively

**Fig. 4:**
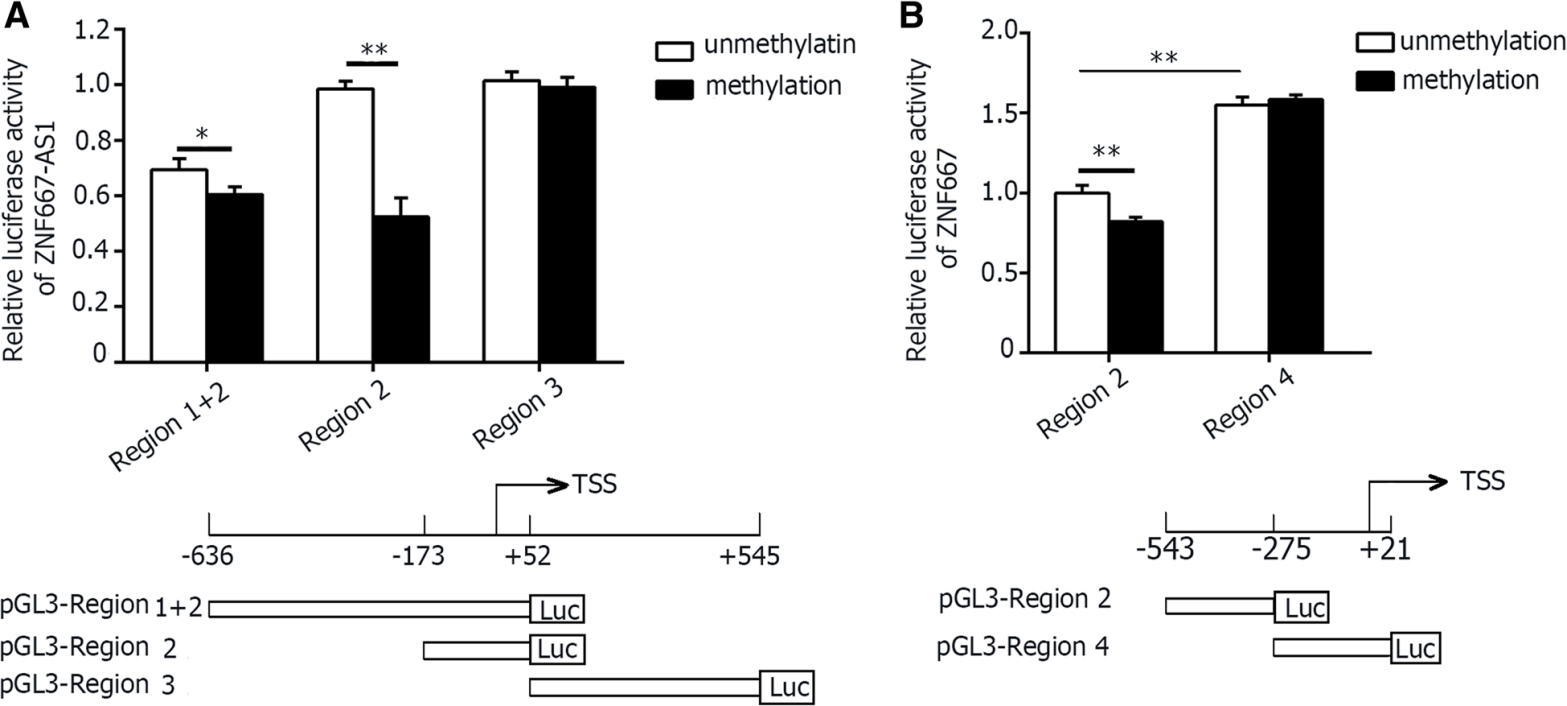
Promoter hypermethylation silences expression of ZNF667-AS1 or ZNF667. **a**. Promoter activity of different fragments of ZNF667-AS1 (− 636/+ 52, − 173/+ 52, and + 53/+ 545). The luciferase activity of pGL3- region 2 vector was set to 1. In vitro methylation of region 2 led to a significant decrease in luciferase activity of ZNF667-AS1. The schematic structures of the promoter fragments were listed below. **b**. Promoter activity of different fragments of ZNF667 (− 543/− 275, and − 270/21). The luciferase activity of pGL3- region 2 vector was set to 1. In vitro methylation of region 2 led to a significant decrease in luciferase activity of ZNF667. The schematic structures of the promoter fragments were listed below. *P < 0.05, **P < 0.01

In UCSC, ZNF667-AS1 and ZNF667 were located in the same chromosome, 19q13.43, transcripting in reverse direction. The two genes were at a distance of 473 bp, where was just their regulation region, as shown in Fig. 1a. For analyzing regulation role of methylation in the expression of ZNF667-AS1 and ZNF667, three reporter constructs were designed for ZNF667-AS1 promoter, and two for ZNF667. Among unmethylated DNA fragments, pGL3-region 2 and pGL3-region 3 had the same promoter activity, both greater than that of pGL3-region 1 + 2. Among methylated DNA fragments, pGL3-region 2, which harbored sequences from − 173 to + 52 bp with respect to the TSS of ZNF667-AS1, had the greatest reduced transcription of a luciferase reporter plasmid (Fig. 4a). The pGL3-region 2 of ZNF667 (− 543 to − 275 bp with respect to the TSS of ZNF667) harbored the same CpG sites as pGL3-region 2 of ZNF667-AS1. Methylation of pGL3-region 2 of ZNF667 also reduced luciferase expression, while the downstream, pGL3-region 4, did not reduce luciferase expression (Fig. 4b). This implied that hypermethylation of promoter decreased the expression level of both ZNF667-AS1 and ZNF667. Here, the downstream of ZNF667 had greater luciferase reporter activity, indicating that ZNF667 was also regulated by other factors.

### Cells with overexpression of ZNF667-AS1 or ZNF667 displayed decreased proliferation and clone formation efficiency, as well as decreased invasion and migration capabilities

**Fig. 5:**
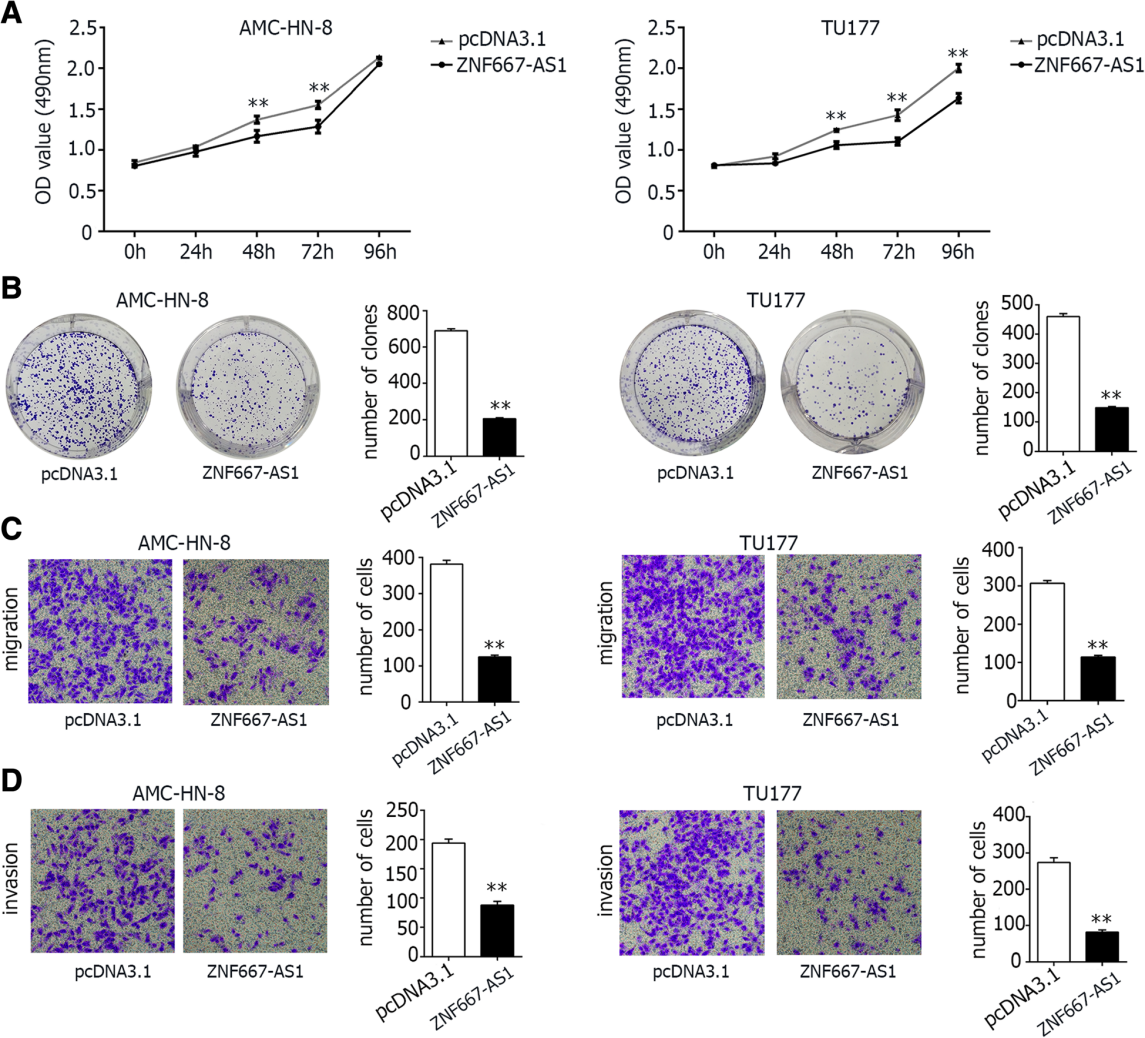
In vitro function of AMC-HN-8 and TU177 cells with overexpression of ZNF667-AS1. **a**. MTS assays of cells transfected with pcDNA3.1 empty vector or pcDNA3.1-ZNF667-AS1, showing that cell transfected with pcDNA3.1-ZNF667-AS1 proliferate slower than that transfected with empty vector at the indicated time points. **b**. Clone formation assays of cells with or without overexpression of ZNF667-AS1. Cells overexpressing ZNF667-AS1 formed less clones than cells transfected with pcDNA3.1 empty vector. **c**. Transwell migration assays of AMC-HN-8 and TU177 cells were performed with or without ZNF667-AS1 overexpression. The migration ability of cells overexpressing ZNF667-AS1 decreased than cells transfected with pcDNA3.1 empty vector. **d**. Transwell invasion assays of AMC-HN-8 and TU177 cells were performed with or without ZNF667-AS1 overexpression. The invasion ability of cells overexpressing ZNF667-AS1 decreased than cells transfected with pcDNA3.1 empty vector. pcDNA3.1: the vector control group; ZNF667-AS1: ZNF667-AS1 over-expression group. The difference was statistically significant, **P < 0.01

**Fig. 6:**
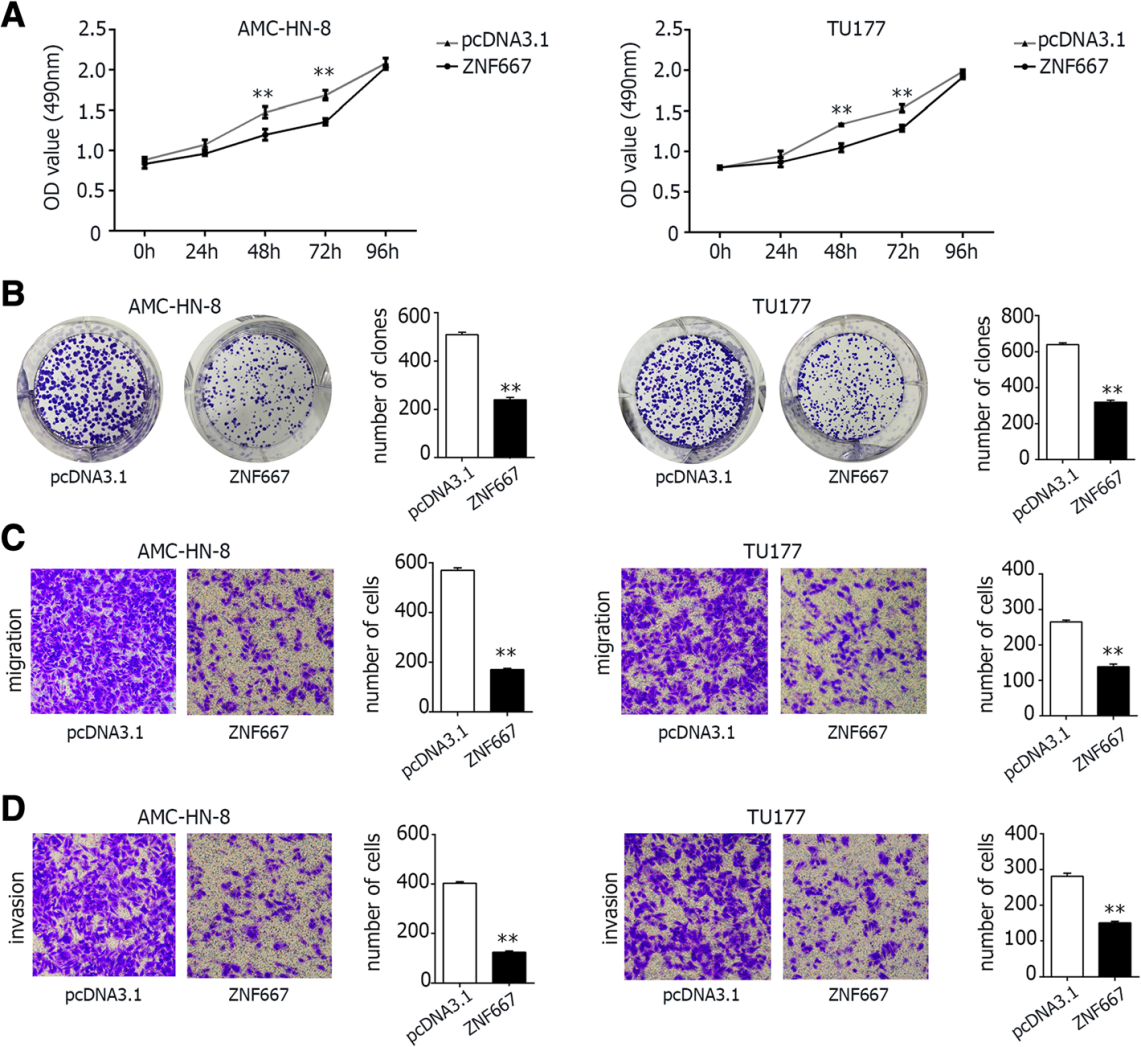
In vitro function of AMC-HN-8 and TU177 cells with overexpression of ZNF667. **a**. MTS assays of cells transfected with pcDNA3.1 empty vector or pcDNA3.1-ZNF667, showing that cell transfected with pcDNA3.1-ZNF667 proliferate slower than that transfected with empty vector at the indicated time points. **b**. Clone formation assays of cells with or without overexpression of ZNF667. Cells overexpressing ZNF667 formed less clones than cells transfected with pcDNA3.1 empty vector. G. Transwell migration assays of AMC-HN-8 and TU177 cells were performed with or without ZNF667 overexpression. The migration ability of cells overexpressing ZNF667 decreased than cells transfected with pcDNA3.1 empty vector. H. Transwell invasion assays of AMC-HN-8 and TU177 cells were performed with or without ZNF667 overexpression. The invasion ability of cells overexpressing ZNF667 decreased than cells transfected with pcDNA3.1 empty vector. pcDNA3.1: the vector control group; ZNF667: ZNF667 over-expression group. The difference was statistically significant, **P < 0.01

AMC-HN-8 and TU177 cells, 24 h after transfected with pcDNA3.1- ZNF667- AS1, pcDNA3.1-ZNF667, or pcDNA3.1 empty vector, were used to investigate biological traits of ZNF667- AS1 or ZNF667. As shown in Figs. 5a and 6a, the cells transfected with pcDNA3.1- ZNF667- AS1 or pcDNA3.1-ZNF667 had decreased proliferation capability than cells transfected with empty vectors, determined by MTS assay. As shown in Figs. 5b and 6b, reduced colony formation was found in cells with overexpression of ZNF667- AS1 or ZNF667, proved by colony formation assay. As shown in Figs. 5c, d, 6c and d, cells with overexpression of ZNF667- AS1 or ZNF667 displayed statistically reduced migration and invasion capabilities in transwell migration and invasion assay. These results displayed that enhancing ZNF667-AS1 or ZNF667 in AMC-HN-8 or TU177 could significantly reduced their proliferation, clone formation, migration and invasion capabilities.

### Overexpression of ZNF667-AS1 increased the expression of ZNF667

**Fig. 7:**
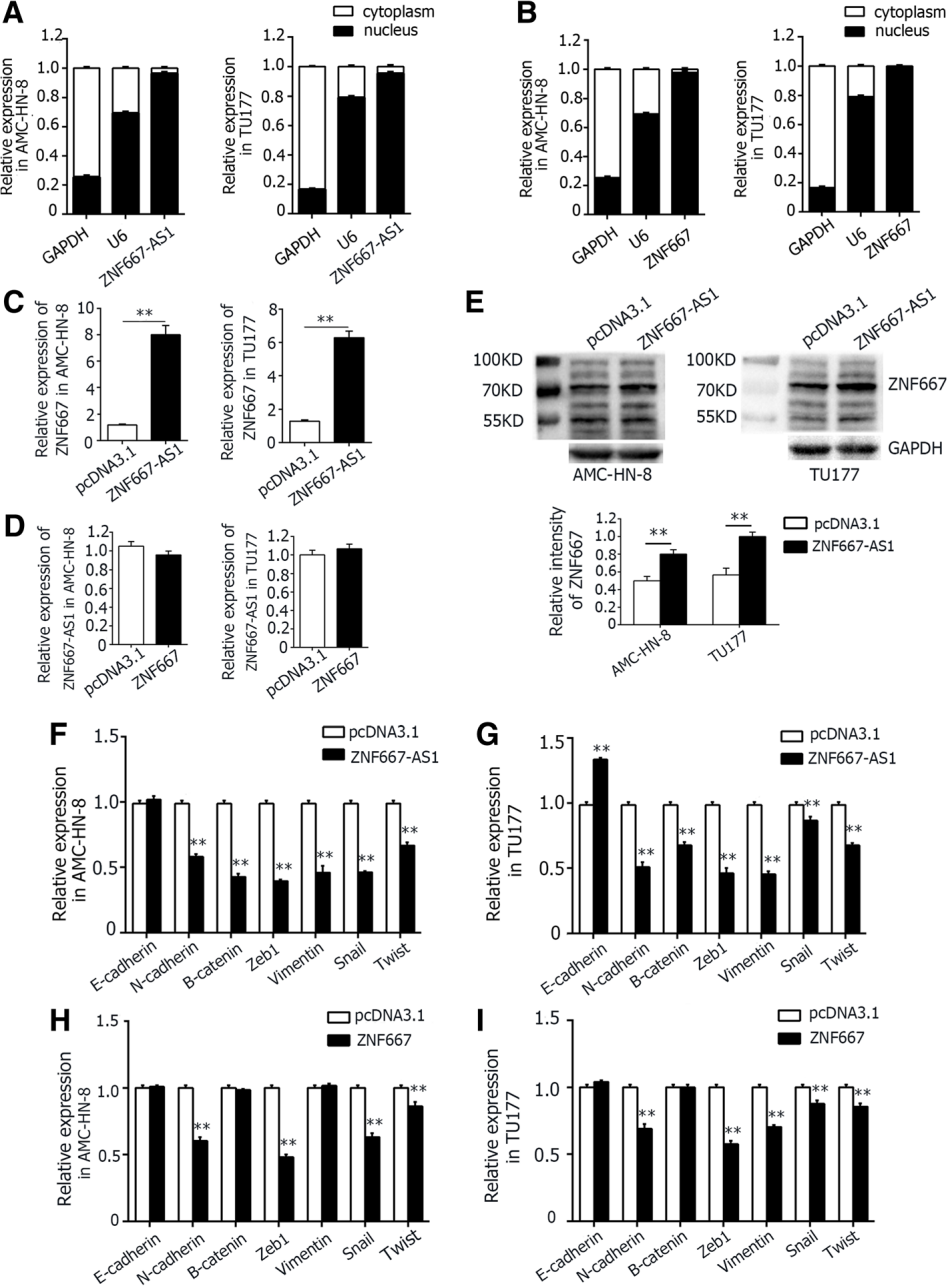
The regulation mechanism of ZNF667-AS1 and ZNF667. **a**. The RNA of ZNF667-AS1 was mainly located on the nucleus. **b**. The mRNA of ZNF667 was mainly located on the nucleus. **c**. Overexpression of ZNF667-AS1 increased the expression of ZNF667 in mRNA level, detected by qRT-PCR. **d**. Overexpression of ZNF667 didn’t increase the expression of ZNF667-AS1 in mRNA level, detected by qRT-PCR. **e**. Overexpression of ZNF667-AS1 increased the expression of ZNF667 in protein level, detected by western blot. **f** and **g**. Overexpression of ZNF667-AS1 increased the expression of EMT associated markers. **h** and **i**. Overexpression of ZNF667 increased the expression of EMT associated markers partially. In Fig. 7c-i, pcDNA3.1: the vector control group; ZNF667-AS1: ZNF667-AS1 over-expression group; ZNF667: ZNF667 over-expression group. The difference was statistically significant, **P < 0.01

As shown in Fig. 7a and b, the RNAs of ZNF667-AS1 and ZNF667 were all mainly located in nucleus in AMC-HN-8 and TU177. As shown in Fig. 7c, AMC-HN-8 and TU177 cells, transfected with pcDNA3.1-ZNF667-AS1, expressed high level of ZNF667 in mRNA level. But AMC-HN-8 and TU177 cells transfected with ZNF667 didn’t display increased expression level of ZNF667-AS1 (Fig. 7d). The protein expression level of ZNF667 was further detected by western blot assay in ZNF667-AS1 transfected AMC-HN-8 or TU177 cells. As shown in Fig. 7e, the cancer cells with overexpression of ZNF667-AS1 expressed higher protein level of ZNF667 compared with cells transfected with empty vector. Therefore, ZNF667-AS1 may regulate the expression of ZNF667.

### Up-regulation of ZNF667-AS1 or ZNF667 led to EMT

EMT was an important way for epithelial cell to get the ability of migration, as well as primary invasion and distant metastasis, by endowing cells with a more motile, invasive potential. To further investigate the molecular mechanism, EMT associated markers were detected. As shown in Fig. 7f and g, both AMC-HN-8 and TU177 cells transfected with pcDNA3.1-ZNF667-AS1 demonstrated decreased expression level of EMT associated molecular markers (N-cadherin, β-catenin, Zeb1, vimentin, snail, and twist), and TU177 also showed increased expression level of E-cadherin. As shown in Fig. 7h and i, the expression of N-cadherin, Zeb1, snail, and twist were lower in ZNF667 transfected AMC-HN-8 and TU177 cells, and vemintin was also lower in TU177.

## Discussion

Dysregulation of long noncoding RNAs play important roles in the development of tumor, and aberrant methylation is one of the most frequent epigenetic alterations in tumors. Increasing evidence has emerged to suggest that ZNF667-AS1 is down-regulated in various tumors [8–11], and 5-aza-2′-deoxycytidine reactivated ZNF667-AS1 expression [8]. By bioinformatic analysis of TCGA data, epigenetic silencing of lncRNA ZNF667-AS1 was found in 16 TCGA cancer types [11], including HNSC (Head and neck squamous cell carcinoma). Here, we found the expression of ZNF667-AS1 in LSCC was also decreased. Treatment with 5-Aza-dC increased the expression level in laryngeal cancer cell lines. The expression was negatively correlated with methylation. The reduced expression or the increased methylation of ZNF667-AS1 in tumor tissues was associated with moderate/poor pathological differentiation. Lukas Vrba, et al. reported that epigenetic silencing of ZNF667-AS1 was an early event in cancer which maintained throughout progression towards the invasive and metastatic disease [9]. And ZNF667-AS1 methylation did not appear to increase further during progression from ductal carcinoma in situ to invasive breast cancer, nor during progression to the metastatic disease [9]. It was reported that ZNF667-AS1 had inhibitory function on the proliferation of cervical cancer, and the expression of ZNF667-AS1 was negatively correlated with the overall survival of cervical cancer [10]. We found that the low expression of ZNF667-AS1 was correlated with moderate/poor pathological differentiation, suggesting ZNF667-AS1 as a tumor suppressor gene in LSCC. In support of this view, those with low expression of ZNF667-AS1 in HNSC tend to had poor survival rate, as indicated by GEPIA (Additional file 2: Figure S1C). It was reported that low-expressed ZNF667-AS1 was an independent prognostic factor for cervical cancer [10]. The prognostic information was needed to further evaluate whether ZNF667-AS1 expression was correlated with LSCC patients’ survival.

The sense transcript, named ZNF667, is a 3877 bp polyadenylated RNA, and often acts as a transcriptional repressor [13, 14]. ZNF667-AS1 gene does not share homology with ZNF genes although located within a ZNF gene cluster. ZNF667-AS1 and ZNF667 share the same CpG islands in the region upstream of the transcriptional start site of the sense and antisense transcripts. Although numerous studies have focused on the analysis of the expression patterns of lncRNAs and their possible crosstalk with adjacent protein-coding genes, little is known about ZNF667-AS1 and ZNF667. The expression level of ZNF667 in various human nomal tissues are consistent with ZNF667-AS1 in the present study. Lukas Vrba et al. reported that the expression of ZNF667 was downregulated during immortalization in human mammary epithelial cells [8]. We found ZNF667 was also downregulated in LSCC. To investigate the potential crosstalk between sense and antisense transcripts, we monitored ZNF667-AS1 and ZNF667 mRNA by qRT-PCR in 47 pairs of LSCC tissues, and a positive correlation between them was observed, suggesting that sense and antisense transcripts are functionally linked. In support of this view, we found that laryngeal cancer cells with overexpression of ZNF667-AS1 displayed decreased proliferation, invasion, and migration capabilities. Cells with overexpression of ZNF667 had the same biological traits, indicating the tumor suppressor role of ZNF667. Those with low expression of ZNF667 in HNSC tend to had poor survival rate, as indicated by GEPIA (Additional file 2: Figure S1D), with the same trend of ZNF667-AS1. However, Ke Cheng et al. reported that ZNF667 was up-regulated in hepatocellular carcinoma and served as a putative oncogene [15]. Given genes are expressed in a cell- and tissue-specific manner, the different roles of ZNF667 in different cancers may be explained by the different basic expression level in normal tissues. As indicated by NCBI, the expression of ZNF667 is low in liver (Fig. 2a).

The tumor suppressor genes were usually silenced by aberrant DNA hypermethylation. In human leukemia, promoter methylation of Idb4, a tumor suppressor gene, led to gene silencing [19]. In this study, we found the expression of both ZNF667-AS1 and ZNF667 were reactivated by 5-aza-2′-deoxycytidine, and hypermethylation of their promoter could reduce the luciferase reporter activity, supporting the notion that their transcriptions are regulated by promoter hypermethylation. The CpG islands in the promoter region of ZNF667-AS1 or ZNF667 were mainly methylated compared with normal tissues. In this study, we explore the mythylation status of ZNF667-AS1 intensively. The hypermethylation in the CpG sites closing to the transcriptional start site (TSS) was associated with reduced expression level of ZNF667-AS1, and was critical for gene silencing. Just like lncRNA LOC100130476, which had three CpG islands in its upstream, aberrant hypermethylation of the CpG sites closing to the TSS was more critical for gene silencing [20]. Insights into the molecular mechanisms that regulate ZNF667-AS1 and ZNF667 expression may therefore unravel new directions of diagnostic and therapeutic strategies. The methylation of DNA can be detected in body fluid, and methylation status of salivary DNA was performed for early detection of head and neck squamous cell carcinoma [21, 22]. Methylation of ZNF667-AS1 and ZNF667 might be used as molecular markers for the early detection of LSCC.

ZNF667-AS1 and ZNF667 are all polyadenylated RNA, indicating that they are all amplified by RNA polymerase II. To avoid collision of RNA polymerases, sense and antisense transcription should not occur simultaneously. Anna Postepska-Igielska, et al. reported that, as to Khps1 and Sphk1, the synthesis of antisense RNA precedes transcription of sense RNA [23]. Importantly, we found overexpression of ZNF667-AS1 led to the increased expression of ZNF667, and overexpression of ZNF667 didn’t change the expression of ZNF667-AS1, indicating that antisense lncRNA, ZNF667-AS1, exert regulation function on sense RNA, ZNF667. The luciferase activity of ZNF667 promoter fragment pGL3-region 4, closing to TSS, was greater than the other, indicating ZNF667-AS1 might exert function through this region.

It was well recognized that enhanced cell proliferation, migration, and invasion state of LSCC cells plays key roles in the progression of LSCC [24, 25]. Strikingly, cellular proliferation, migration, and invasion was decreased following ectopic expression of ZNF667-AS1 in AMC-HN-8 and TU177 cells. To further confirm whether overexpression of ZNF667 inhibits proliferation, clonability, migratory and invasion potential, we transfected ZNF667 in AMC-HN-8 and TU177 cells. Elevated ZNF667 levels reduced the growth, migration, and invasion of cancer cells in vitro. All of the above indicated ZNF667-AS1 and ZNF667 as tumor suppressor genes in LSCC carcinogenesis.

To initiate the metastatic process, cancer cells must first penetrate the epithelial basement membrane. The degradation of extracellular matrix (ECM) is associated with cell invasion. In the present study, the ZNF667-AS1 or ZNF667 transfected cells penetrated less through the Matrigel as compared with the control cells. It appeared that the overexpression of ZNF667-AS1 or ZNF667 attenuated the ability for cells to digest the matrix molecules, which decreased cell invasion and metastasis. To investigate probable underlying mechanisms, EMT associated markers were detected. EMT associated molecular markers (N-cadherin, β-catenin, Zeb1, vimentin, snail, twist) were lower in cells transfected with ZNF667-AS1, and E-cadherin even was higher in TU177. There was similar trend in ZNF667 overexpression cells, but the changed markers were fewer. Taken together, these findings indicated that ZNF667-AS1 or ZNF667 decreased cancer cell migration and invasion in vitro. Cells with downregulated ZNF667-AS1 or ZNF667 may undergo epithelial-mesenchymal transition (EMT) process, becoming more mobile and invasive, and promote the malignant progression of LSCC.

## Conclusions

In conclusion, ZNF667-AS1 and ZNF667 were both downregulated by methylation, and both served as tumor suppressor genes in LSCC. ZNF667-AS1 regulated the expression of ZNF667 in cis.

## Additional files

Additional file 1: **Table S1.** Primer sequences, annealing temperature and product size of ZNF667-AS1 and ZNF667 gene Family. (DOCX 20 kb)

Additional file 2: **Figure S1.** Supplementary data. A. The representative methylation status of three regions of ZNF667 detected by MSP analysis in LSCC patients. B. Relative expression of ZNF667 in the tumor tissues with and without methylation of the three regions, which was expressed as mean ± S.D. * *P* < 0.05. C. Survival analysis of ZNF667-AS1 in LSCC, cited from GEPIA. D. Survival analysis of ZNF667 in LSCC, cited from GEPIA. (TIF 531 kb)
