# Supplementary material for: Aberrant methylation and downregulation of ZNF667-AS1 and ZNF667 promote the malignant progression of laryngeal squamous cell carcinoma
Source: J Biomed Sci. 2019 Jan 26;26:13. doi: 10.1186/s12929-019-0506-0 (PMC6347788; doi:10.1186/s12929-019-0506-0)
Supplement: Supplementary file 1 — Table S1. Primer sequences, annealing temperature and product size of ZNF667-AS1 and ZNF667 gene Family. (DOCX 20 kb) [file 12929_2019_506_MOESM1_ESM.docx]

**Table S1. Primer sequences, annealing temperature and product size of** **ZNF667-AS1 and ZNF667 gene Family**

| Types | Primer sequence | Annealing temperature (℃) | Product size(bp) |
| --- | --- | --- | --- |
| ZNF667-AS1 |  |  |  |
| Region1-methylation | F: 5’- TGGGTTACGTAGCGATATATATTCG -3’ | 48 | 182 |
|  | R:5’- TAAACTCACGCCTACGTAAATTCTCG -3’ |  |  |
| Region1-unmethylation | F: 5’- TGGGTTATGTAGTGATATATATTTG -3’ | 48 | 182 |
|  | R:5’- TAAACTCACACCTACATAAATTCTCA -3’ |  |  |
| Region2-methylation | F: 5’- TCGTTTTTATAATTATACGTTTTCG -3’ | 55 | 155 |
|  | R:5’- AATCGCAAAATAACTACGTAATACG -3’ |  |  |
| Region2-unmethylation | F: 5’- TTGTTTTTATAATTATATGTTTTTG -3’ | 51 | 155 |
|  | R: 5’- AATCACAAAATAACTACATAATACA -3’ |  |  |
| Region3-methylation | F: 5’- TTTATTTCGGCGGGGAGAAGGGACG -3’ | 59 | 154 |
|  | R: 5’- CCCGCACGCCCAAATAACCGAAACG -3’ |  |  |
| Region3-unmethylation | F: 5’- TTTATTTTGGTGGGGAGAAGGGATG -3’ | 59 | 154 |
|  | R: 5’- CCCACACACCCAAATAACCAAAACA -3’ |  |  |
| BGS region 1 | F: 5’- GTTAATTTTATATTTGGG -3’ | 46 | 450 |
|  | R: 5’- AAACTAATAATACTATCCC -3’ |  |  |
| BGS region 2 | F: 5’- AGTTTTTTAATTAAATTTAATTT -3’ | 48.5 | 271 |
|  | R: 5’- CAACCCAAACCTTCTTCACCTCA -3’ |  |  |
| BGS region 3 | F: 5’- GGTTGGTAGTGAGATTGATTG -3’ | 51.6 | 271 |
|  | R: 5’- TCCTCACACATATTCAAAATAAC -3’ |  |  |
| pGL3- region 1+2 | F: 5’- TACAGATCTTCAGACACACCCACACTC -3’ | 63 | 688 |
|  | R: 5’- TATAAGCTTCTCACGTTCTGGGTGGG -3’ |  |  |
| pGL3- region 3 | F: 5’- GATAGATCTGTGAAGAAGGCTTGGGCT -3’ | 58 | 493 |
|  | R: 5’- TGCAAGCTT CTCACACATGTTCAGGGT -3’ |  |  |
| qRT-PCR | F: 5’- GGGAGTGTCCGCCATAAAGT -3’ | 62 | 133 |
|  | R: 5’- CTACACAAACGCGCGATCAA -3’ |  |  |
| ZNF667 |  |  |  |
| Region1-methylation | F: 5’- TTCGCGTTTGCGTGGGTTTTCG-3’ | 68 | 178 |
|  | R: 5’- TAAATCACGCAACGATACACATCCG-3’ |  |  |
| Region1-unmethylation | F: 5’- TTTGTGTTTGTGTGGGTTTTTG -3’ | 62 | 178 |
|  | R: 5’- TAAATCACACAACAATACACATCCA -3’ |  |  |
| Region2-methylation | F: 5’- GGTCGTAGGATGATTGCGTAGTGCGTA -3’ | 65 | 154 |
|  | R: 5’- CGCTCCCACAACCACACGTTCCCGTATA -3’ |  |  |
| Region2-unmethylation | F: 5’- GGTTGTAGGATGATTGTGTAGTGTGTA -3’ | 61 | 154 |
|  | R: 5’- CACTCCCACAACCACACATTCCCATATA -3’ |  |  |
| Region3-methylation | F: 5’- TTCGTACGTTTAGATGATCGGGACG-3’ | 58 | 161 |
|  | R: 5’- AATAAAACTCACCCCGACGAA -3’ |  |  |
| Region3-unmethylation | F: 5’- TTTGTATGTTTAGATGATTGGGATG -3’ | 58 | 161 |
|  | R: 5’- AATAAAACTCACCCCAACAAA -3’ |  |  |
| BGS region 1 | F: 5’- AGAGTGTGGGTGTGTTTGAG -3’ | 56 | 379 |
|  | R: 5’- AACACAAATCTTCACAATCAAC -3’ |  |  |
| BGS region 2 | F: 5’- GGTGGATTTTGGAGGT -3’ | 56 | 208 |
|  | R: 5’- CTCTTTAACCAAACCCAAC -3’ |  |  |
| BGS region 3 | F: 5’- TAATATAATGTGGGTTTTATTTAT -3’ | 50 | 252 |
|  | R: 5’- AACTAACAATAAAACTAACTAC -3’ |  |  |
| pGL3- region 2 | F: 5’- GATAGATCTCAGCCCAAGCCTTCTTCACCT -3’ | 58 | 286 |
|  | R: 5’- GTGAAGCTTTCTTTAACCAAACCCAACCTCC -3’ |  |  |
| pGL3- region 4 | F: 5’- GACAGATCTAGGAAGGGCCTCAGTGTCC -3’ | 67.4 | 309 |
|  | R: 5’- GATAAGCTTCTTCACACACGCACCACGCA -3’ |  |  |
| qRT-PCR | F: 5’- TGTGACAAGTTCTTCAGGCG -3’ | 60 | 114 |
|  | R: 5’- GGATGAATGCCGATTGCAGAC -3’ |  |  |
| Control genes |  |  |  |
| GAPDH | F: 5’- AGGTGAAGGTCGGAGTCAACG -3’ | 58 | 102 |
|  | R: 5’- AGGGGTCATTGATGGCAACA -3’ |  |  |
| U6 | F: 5’- GCTTCGGCAGCACATATACTAAAAT -3’ | 58 | 89 |
|  | R: 5’- CGCTTCACGAATTTGCGTGTCAT -3’ |  |  |
