# Supplementary figures and images for: Aberrant methylation and downregulation of ZNF667-AS1 and ZNF667 promote the malignant progression of laryngeal squamous cell carcinoma
Source: J Biomed Sci. 2019 Jan 26;26:13. doi: 10.1186/s12929-019-0506-0 (PMC6347788; doi:10.1186/s12929-019-0506-0)

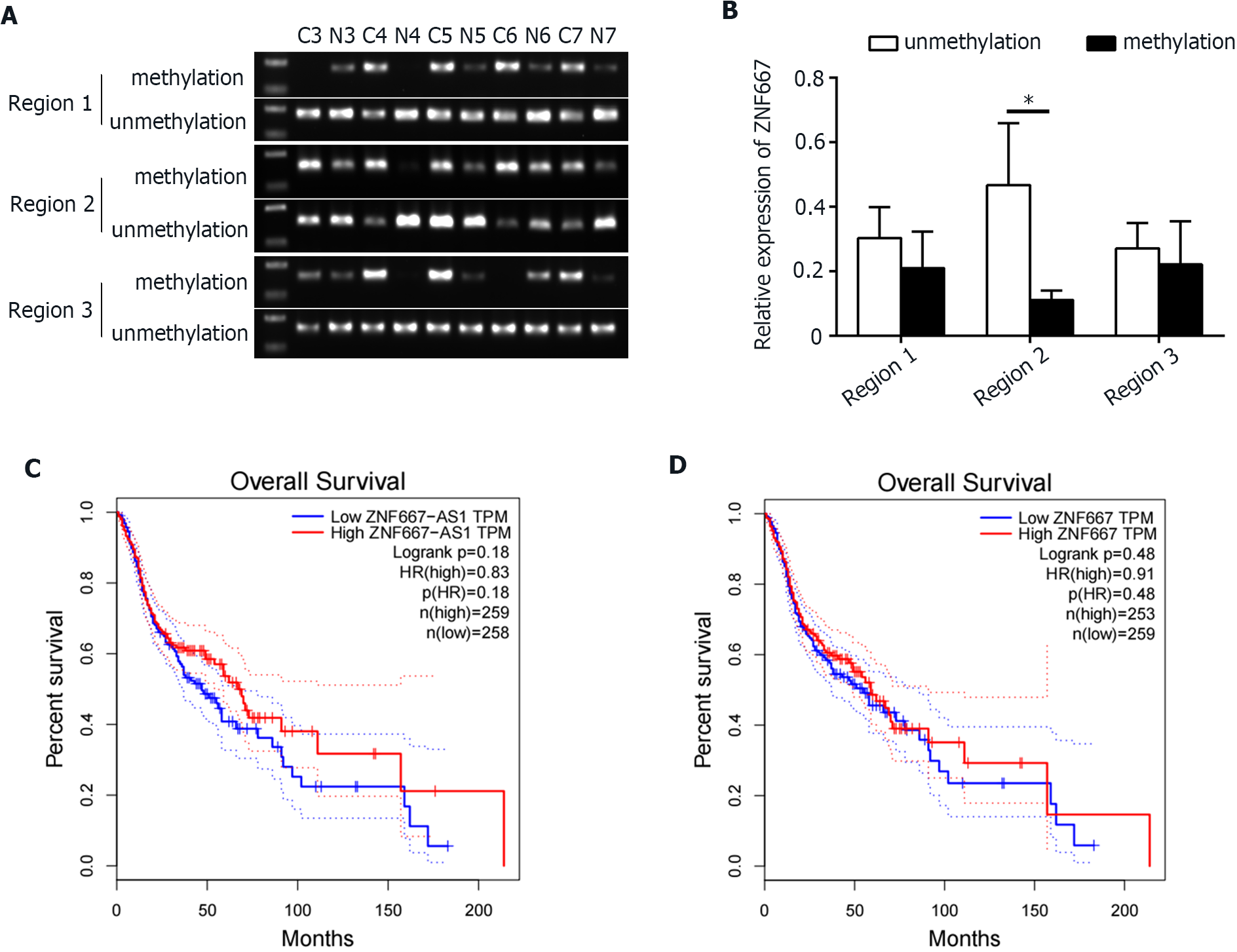

Supplement: Supplementary file 2 — Figure S1. Supplementary data. A. The representative methylation status of three regions of ZNF667 detected by MSP analysis in LSCC patients. B. Relative expression of ZNF667 in the tumor tissues with and without methylation of the three regions, which was expressed as mean ± S.D. * P < 0.05. C. Survival analysis of ZNF667-AS1 in LSCC, cited from GEPIA. D. Survival analysis of ZNF667 in LSCC, cited from GEPIA. (TIF 531 kb) [file 12929_2019_506_MOESM2_ESM.tif]
